# Methamphetamine-Induced Loss of Syndecan-1 and Retinal Endothelial Integrity via the TAAR-1/MMP-9 Pathway

**DOI:** 10.3390/pathophysiology32030041

**Published:** 2025-08-26

**Authors:** Minsup Lee, Taekyung Ha, Ivan A. Alvarez, Wendy Leskova, Changwon Park, Norman R. Harris

**Affiliations:** 1 Department of Molecular and Cellular Physiology, Louisiana State University Health Shreveport, 1501 Kings Hwy, Shreveport, LA 71103, USA; minsup.lee@lsuhs.edu (M.L.); taekyung.ha@lsuhs.edu (T.H.); wendy.leskova@lsuhs.edu (W.L.); changwon.park@lsuhs.edu (C.P.); 2 School of Medicine, Louisiana State University Health Shreveport, 1501 Kings Hwy, Shreveport, LA 71103, USA; ivan.alvarez@uky.edu; 3 Current: Department of Otolaryngology-Head and Neck Surgery, University of Kentucky College of Medicine, 740 S. Limestone Street, Lexington, KY 40536, USA

**Keywords:** methamphetamine, retinal endothelial cell, syndecan-1, matrix metalloproteinase-9, trace amine-associated receptor-1

## Abstract

Background/Objectives: Methamphetamine (METH), a potent psychostimulant, exerts harmful effects on the vascular system by promoting oxidative stress, inflammation, and endothelial injury. While its impact on the blood–brain barrier is well documented, its influence on the retinal microvasculature remains less understood. This study investigated the effects of METH on syndecan-1 expression and endothelial function in primary rat retinal microvascular endothelial cells (RRMECs) and isolated ophthalmic arteries. Methods: We assessed METH-induced changes in mRNA and protein expression levels of syndecan-1, matrix metalloproteinase (MMP)-2, and MMP-9. Endothelial function was evaluated using scratch migration assays and trans-endothelial electrical resistance (TEER) measurements. The mechanistic involvement of MMP-9 and trace amine-associated receptor 1 (TAAR-1), a known receptor for METH, was examined using selective pharmacological inhibitors. Results: METH exposure significantly decreased syndecan-1 expression and increased MMP-9 levels. These changes were accompanied by impaired endothelial migration and reduced TEER in RRMECs. Similar findings were confirmed in cultured ophthalmic arteries, reinforcing the translational relevance of our in vitro results. Inhibition of MMPs restored syndecan-1 expression and rescued endothelial function. Furthermore, TAAR-1 antagonism protected against syndecan-1 degradation, reduced MMP-9 upregulation, and improved endothelial migration and barrier resistance. Conclusions: Our findings suggest that METH induces loss of syndecan-1 and retinal vascular integrity by promoting TAAR-1–mediated MMP-9 upregulation. Targeting the TAAR-1/MMP-9 axis may offer a promising therapeutic strategy for preventing METH-induced microvascular damage in the retina.

## 1. Introduction

Methamphetamine (METH) significantly impacts circulatory dynamics by influencing vascular tone, cardiac function, and endothelial health. Acting as a potent sympathomimetic agent, METH elevates catecholamine levels, triggering vasoconstriction, increased arterial pressure, and tachycardia—factors that contribute to hypertension and cardiac strain [1]. Chronic METH use further exacerbates vascular dysfunction through oxidative stress and inflammation, leading to endothelial injury [1,2].

Importantly, METH profoundly disrupts the blood–brain barrier (BBB), a selectively permeable interface that protects neural tissue from circulating toxins and inflammatory cells. METH-induced oxidative stress and matrix metalloproteinase (MMP) activation degrade tight junction proteins such as claudins and occludins, resulting in increased BBB permeability [3,4]. The blood–retina barrier (BRB), which shares structural and functional similarities with the BBB, is also highly sensitive to oxidative and inflammatory insults and may be similarly compromised by psychostimulants [5,6]. Given the essential roles of the BBB and BRB in maintaining neural homeostasis, their disruption by METH represents a key mechanism of neuronal and retinal injury.

In addition, METH alters cerebral and retinal blood flow [7] and degrades endothelial surface molecules, including proteoglycans [2], which are critical components of the glycocalyx and endothelial permeability regulation. Glycocalyx degradation may further destabilize vascular integrity and contribute to microvascular dysfunction [8,9]. These pathological effects may explain the increased risk of vascular events—such as brain or retinal ischemia—in chronic METH users [10]. Since the glycocalyx plays a central role in vascular stability, its compromise may exacerbate both neurological and retinal pathologies. Therefore, strategies aimed at preserving or restoring glycocalyx integrity may provide promising therapeutic avenues for mitigating METH-induced complications in the central nervous system and eye.

Syndecan-1 is a transmembrane heparan sulfate proteoglycan expressed on endothelial cells, where it plays a crucial role in vascular homeostasis [11]. As a core component of the endothelial glycocalyx, syndecan-1 regulates vascular permeability, mechanotransduction, and inflammatory responses. Through its heparan sulfate chains, it binds and modulates the activity of growth factors, cytokines, and extracellular matrix proteins, thereby influencing angiogenesis, endothelial repair, and leukocyte–endothelium interactions [12,13,14]. Under pathological conditions—such as inflammation, oxidative stress, ischemia, or hyperglycemia—syndecan-1 is cleaved from the endothelial surface by MMPs and A disintegrin and metalloproteinases (ADAMs) [12,15,16]. This proteolytic removal contributes to glycocalyx degradation, increased endothelial permeability, leukocyte adhesion, and impaired barrier function [17,18].

Loss of syndecan-1 has been implicated in various retinal vascular disorders, including diabetic retinopathy, hypertensive retinopathy, and METH-induced retinopathy [2,12,19]. While syndecan-1 is well-known for its regulatory role in inflammatory responses, it also plays a critical role in regulating endothelial cell migration by modulating receptor–ligand interactions at the cell surface [20]. In pathological conditions such as tumor angiogenesis, diabetic retinopathy, and ischemia, dysregulation of syndecan-1 may lead to aberrant endothelial migration and abnormal vascular remodeling, contributing to disease progression. Thus, targeting syndecan-1 may offer therapeutic potential in modulating endothelial dynamics and improving vascular outcomes.

In this study, we hypothesized that METH induces loss of syndecan-1 in primary rat retinal microvascular endothelial cells (RRMECs) and contributes to vascular dysfunction by impairing endothelial migration and increasing permeability. To test this hypothesis, we examined the effects of METH on syndecan-1 expression in RRMECs and isolated ophthalmic arteries (OA) and assessed its influence on endothelial migration and barrier integrity. Furthermore, we used specific inhibitors to delineate the mechanistic involvement of syndecan-1 degradation and endothelial dysfunction.

## 2. Materials and Methods

### 2.1. Cells and Blood Vessel Tissue Culture

METH was obtained from the Research Triangle Institute (Research Triangle Park, NC, USA) by the National Institute on Drug Abuse Drug Supply Program. Recombinant MMP-9 and EPPTB [N-(3-ethoxyphenyl)-4-(1-pyrrolidinyl)-3-(trifluoromethyl)benzamide)] were purchased from Bio-Techne (Minneapolis, MN, USA). GM6001 was obtained from Millipore-Sigma (Burlington, MA, USA). The RRMECs (Cell Biologics, Chicago, IL, USA) were incubated with or without 2 µM of METH for 3 days. Wistar rats (male) were purchased from Charles River (Wilmington, MA, USA) and maintained under controlled environmental conditions (22 °C, 12-h light/dark cycle). The animal protocols were approved by the Institutional Animal Care and Use Committee of Louisiana State University Health Sciences Center-Shreveport and adhere to the ARVO Statement for the Use of Animals in Ophthalmic and Vision Research. OA beside the optic nerve were collected from rats after euthanization with CO_2_ and washed in phosphate-buffered saline (PBS) containing penicillin (100 unit/mL), streptomycin (100 µg/mL), and Amphotericin B (25 ng/mL) and then incubated in the media containing penicillin (100 unit/mL), streptomycin (100 µg/mL), and Amphotericin B (25 ng/mL) for 1 day. PBS (1:1000, *v*/*v*) was used as a vehicle control.

### 2.2. Immunoblot Analysis

The incubated cells or blood vessels were lysed in radioimmunoprecipitation assay buffer and then centrifuged at 14,000 rpm for 20 min at 4 °C for collecting whole protein lysates. Protein concentrations were determined by the bicinchoninic acid assay (Thermo Fisher Scientific, Waltham, MA, USA). Proteins were denatured and reduced in Laemmli sample buffer (Bio-Rad, Hercules, CA, USA) containing 2.5% β-mercaptoethanol (Bio-Rad) for 10 min at 100 °C. Equal volumes of protein were separated by electrophoresis in 8% sodium dodecyl sulfate-polyacrylamide gel and transferred onto a nitrocellulose membrane. Protein-free T20 blocking solution (Thermo Fisher Scientific) was used for avoiding non-specific binding of antibodies. The membranes were incubated with primary antibodies for MMP-2 (1:1000, *v*/*v*, ab92536, abcam, Cambridge, UK), MMP-9 (1:2000, *v*/*v*, sc-393859, Santa Cruz Biotechnology, Dallas, TX, USA) or syndecan-1 (1:2000, *v*/*v*, ab128936, abcam) overnight. Immune complexes were detected using horseradish peroxidase-conjugated secondary antibodies against rabbit or mouse IgG (Jackson ImmunoResearch Laboratories, West Grove, PA, USA) and Clarity Western enhanced chemiluminescence substrate (Bio-Rad). Images were captured with a Chemidoc Imaging System (Bio-Rad), and densitometric analysis was performed using ImageJ (v1.52a, NIH, Bethesda, MD, USA). β-actin (Santa Cruz Biotechnology) levels from the same membranes were used for normalization of relative protein expression.

### 2.3. Total RNA Isolation, Reverse Transcription, and Quantitative Real-Time Polymerase Chain Reaction (qRT-PCR)

Total RNA was extracted from the incubated cells using a TRIzol lysis reagent (Thermo Fisher Scientific) following the manufacturer’s instructions. Five micrograms of RNA were reverse-transcribed into cDNA using GoScript Reverse transcriptase (Promega, Madison, WI, USA) with an oligo (dT_15_) primer. Quantitative PCR to determine mRNA expression levels was performed on a Bio-Rad CFX Fast Real-Time PCR System with iTaq Universal SYBR Green Supermix (Bio-Rad). Primers are listed in Table 1. Data were analyzed using the 2^−ΔΔCt^ method, with Mapk1 and Peptidylprolyl isomerase A (Ppia) serving as internal controls.

### 2.4. Scratch Cell Migration Assay

Monolayers of RRMECs incubated in 6-well plates for 2 days with or without METH were scraped in a straight line with a p1000 pipet tip. After washing the cells with prewarmed media carefully, images from three areas (including the center) of the scratched monolayer in each well were captured with a phase-contrast microscope (Thermo Fisher Scientific). The cells were further incubated with or without METH for 16 h, and images from the same areas of each well were captured. The average of three distances between the two sides of the scratch from each well were measured by ImageJ and used for comparison.

### 2.5. Transendothelial Electrical Resistance (TEER) Measurement

Retinal endothelial barrier integrity was evaluated using the Maestro Z impedance-based system (Axion BioSystems, Atlanta, GA, USA), according to the manufacturer’s protocol. Briefly, the RRMECs were inoculated onto a CytoView-Z 96-well plate (Axion BioSystems) and incubated under standard conditions (37 °C, 5% CO_2_). Impedance measurements were recorded at 1 kHz using the integrated electrode array in each well. TEER values were automatically calculated by the AxIS Z software (Axion BioSystems) and expressed in Ohms (Ω). The cells were exposed to 2 µM of METH at indicated times to assess its impact on barrier function. TEER values were continuously monitored over a period of 72 h.

### 2.6. Statistics

Statistical analyses were performed using GraphPad Prism 10 software (GraphPad, Boston, MA, USA). Comparisons between two groups were made using unpaired *t*-tests, while comparisons among three or more groups were assessed by one-way analysis of variance followed by Newman–Keuls post hoc tests. Group data are presented as means ± standard error, with *p* < 0.05 considered statistically significant.

## 3. Results

### 3.1. Effect of METH on Syndecan-1 in RRMECs and OA

**Figure 1:**
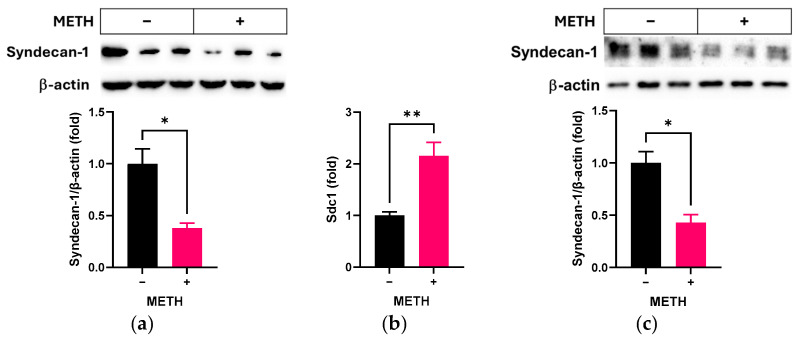
Effect of METH on syndecan-1 in RRMECs and cultured OA. RRMECs and OV were incubated with 2 µM METH for 3 days. (**a**) The relative protein expression levels of syndecan-1 in RRMECs were normalized to β-actin using ImageJ. (**b**) The relative mRNA expression levels of Sdc1 were normalized to Ppia. (**c**) The relative protein expression levels of syndecan-1 in cultured OA were normalized to β-actin using ImageJ. Data are presented as means ± SEM (*n* = 3–6, * *p* < 0.05 and ** *p* < 0.01).

Firstly, we compared the changes in syndecan-1 levels following METH administration in RRMECs and cultured OA. METH led to decreased protein levels of syndecan-1 in both RRMECs (Figure 1a, *n* = 3, *p* < 0.05) and cultured OA (Figure 1c, *n* = 3, *p* < 0.05). However, the mRNA level of syndecan-1 was higher in METH-administered cells compared to the control group (Figure 1b, *n* = 6, *p* < 0.05).

### 3.2. Effect of METH on the Endothelial Cell Migration and Permeability

**Figure 2:**
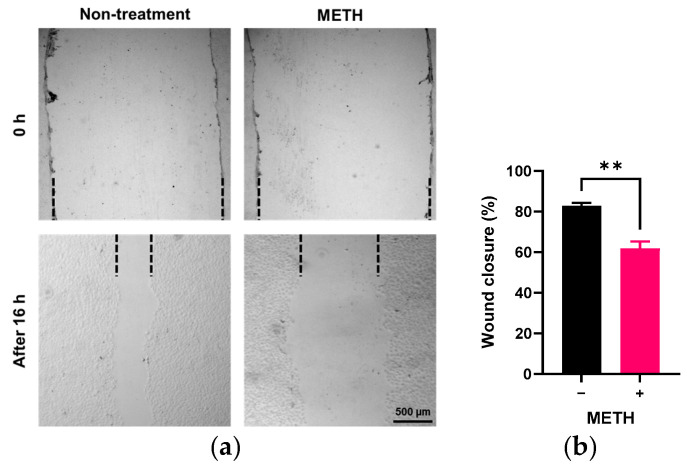
Effect of METH on cell migration in RRMECs. The cells incubated with or without 2 µM for 2 days were further incubated for 16 h after scratch. PBS was used for vehicle control. (**a**) Representative images of scratched areas were captured using a microscope. The dashed line indicates the boundary of the cell monolayer. (**b**) RRMEC migration (“wound closure”) was quantified by measuring the scratched area at 0 h and 16 h using ImageJ. Data are presented as means ± SEM (*n* = 4, ** *p* < 0.01).

To determine whether METH affects endothelial cell migration, we conducted a cell scratch assay. As shown in Figure 2, the recovered area after scratch in the RRMECs incubated with METH for 16 h was significantly lower than in the control group (61.8 ± 3.5% vs. 82.4 ± 1.1%, *n* = 4, *p* < 0.05).

**Figure 3:**
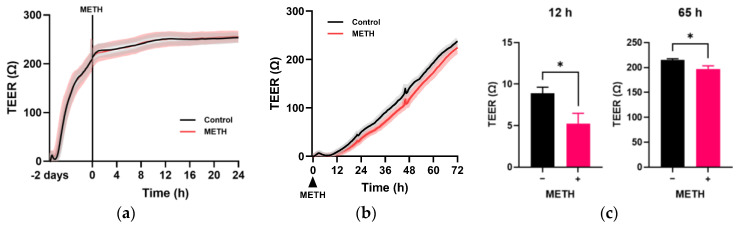
Effect of METH on cell permeability in RRMEC. RRMECs incubated for 48 h were administrated METH for 24 h (**a**), and RRMECs administered METH at time 0 were incubated for 72 h (**b**,**c**). Endothelial barrier function was continuously monitored using a TEER assay (*n* = 4–5, means ± SEM, * *p* < 0.05).

To further investigate the effect of METH on endothelial function, we assessed endo-thelial barrier integrity using a TEER assay. METH administration did not alter the resistance in RRMECs when administered after a stable level had been reached (*n* = 4–6, Figure 3a). However, the resistance of RRMECs administered METH at the initiation stage was significantly lower than control between 12 and 65 h (*n* = 4–5, *p* < 0.05, Figure 3b,c).

### 3.3. Effect of METH on MMP Expression in RRMECs

**Figure 4:**
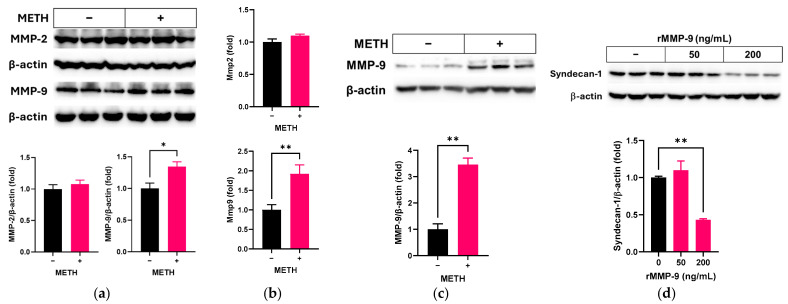
Effect of METH on MMPs in RRMECs and cultured OA. RRMECs and OV were incubated with 2 µM METH for 3 days. (**a**) The relative protein expression levels of MMP-2 and MMP-9 in RRMECs were normalized to β-actin using ImageJ. (**b**) The relative mRNA expression levels of Mmp2 and Mmp9 were normalized to Ppia. (**c**) The relative protein expression levels of MMP-9 in cultured OA were normalized to β-actin using ImageJ. (**d**) The relative protein expression levels of syndecan-1 in RRMECs were normalized to β-actin using ImageJ. Data are presented as means ± SEM (*n* = 3–6, * *p* < 0.05 and ** *p* < 0.01).

Since MMPs can degrade cellular syndecan-1 [16], we examined the potential change in expression of MMPs by METH in RRMECs and cultured OA. In RRMECs, METH administration significantly elevated the expression levels of MMP-9 protein (*n* = 3, *p* < 0.05, Figure 4a) and mRNA (*n* = 6, *p* < 0.01, Figure 4b) compared to control, but not MMP-2 (Figure 4a,b). In cultured OA, MMP-9 protein also was increased by METH administration (*n* = 3, *p* < 0.01, Figure 4c). To confirm whether MMP-9 is associated with loss of syndecan-1 in endothelial cells, RRMECs were incubated with media containing recombinant MMP-9. As shown in Figure 4d, the protein levels of syndecan-1 in RRMECs were significantly decreased in the presence of recombinant MMP-9 compared to control (*n* = 3, *p* < 0.01).

### 3.4. Role of MMP-9 in METH-Induced Disruption of Endothelial Integrity

**Figure 5:**
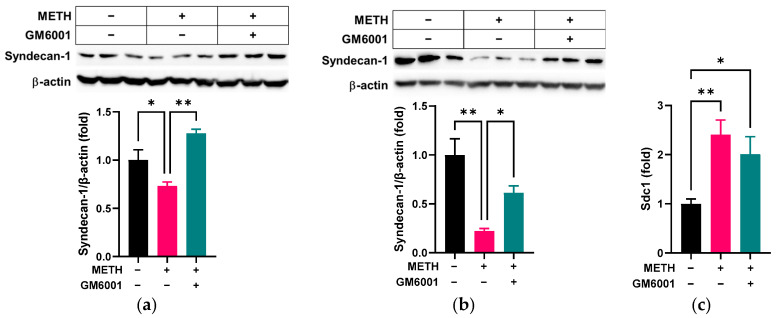
Effect of the MMP inhibitor GM6001 on METH-induced loss of syndecan-1. RRMECs and OV were incubated with 2 µM METH in the presence or absence of 20 µM GM6001 for 3 days. (**a**) The relative protein expression levels of syndecan-1 in RRMECs were normalized to β-actin using ImageJ (*n* = 3, means ± SEM, * *p* < 0.05 and ** *p* < 0.01). (**b**) The relative protein expression levels of syndecan-1 in OA were normalized to β-actin using ImageJ (*n* = 3, means ± SEM, * *p* < 0.05 and ** *p* < 0.01). (**c**) The relative mRNA expression levels of Sdc1 in RRMECs were normalized to Ppia. (*n* = 6, means ± SEM, * *p* < 0.05 and ** *p* < 0.01).

We further investigated whether METH-induced MMP-9 plays a significant role in altering RRMECs and cultured OA by using GM6001, an MMP inhibitor. GM6001 treatment rescued syndecan-1 protein levels in both METH-treated RRMECs (*p* < 0.05, *n* = 3, Figure 5a) and cultured OA (*p* < 0.05, *n* = 3, Figure 5b) without changes of mRNA in RRMECs (Figure 5c), compared to cells administered METH alone.

**Figure 6:**
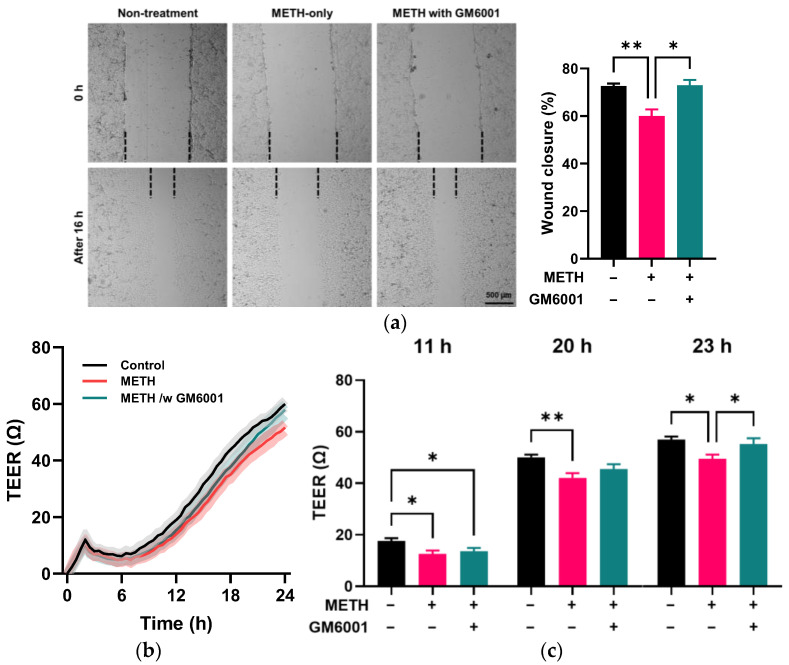
Effect of the MMP inhibitor GM6001 on METH-induced endothelial dysfunction. (**a**) Representative images of scratched areas in RRMECs were captured using a microscope. The dashed line indicates the boundary of the cell monolayer. RRMEC migration (“wound closure”) was quantified by measuring the scratched area at 0 h and 16 h using ImageJ (*n* = 3, means ± SEM, * *p* < 0.05 and ** *p* < 0.01). (**b**,**c**) Endothelial resistance in RRMECs incubated without or with METH in the absence or presence of GM6001 was continuously monitored using a TEER assay (*n* = 6, means ± SEM, * *p* < 0.05 and ** *p* < 0.01).

GM6001 treatment promoted wound closure in METH-administered RRMECs (*p* < 0.05, *n* = 3, Figure 6a) compared to those administered METH-only. In addition, the TEER assay (Figure 6b,c) showed that GM6001 treatment significantly increased resistance in METH-administered RRMECs compared to those administered METH alone (*p* < 0.05, *n* = 6).

### 3.5. Trace Amine-Associated Receptor-1 (TAAR-1)-Mediated Disruption of Endothelial Integrity by METH

**Figure 7:**
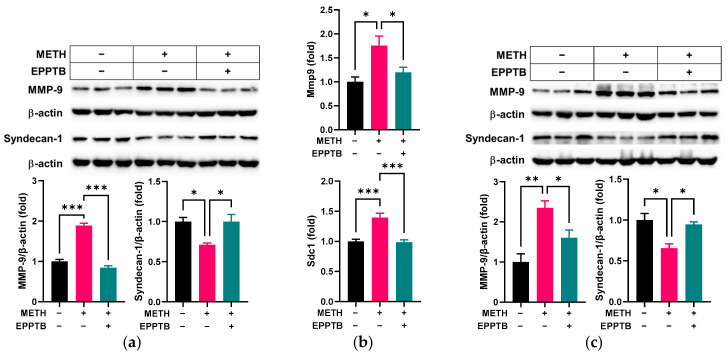
Effect of the TAAR-1 inhibitor, EPPTB, on METH-induced changes. RRMECs and OA were incubated with 2 µM METH in the presence or absence of 0.5 µM EPPTB for 3 days. (**a**) The relative protein expression levels of MMP-9 and syndecan-1 in RRMECs were normalized to β-actin using ImageJ. (**b**) The relative mRNA expression levels of Mmp9 and Sdc1 were normalized to Ppia. (**c**) The relative protein expression levels of MMP-9 and syndecan-1 in cultured OA were normalized to β-actin using ImageJ. Data are presented as means ± SEM (*n* = 3–6, * *p* < 0.05, ** *p* < 0.01, and *** *p* < 0.001).

TAAR-1 is one of the major receptors [21,22] that can be activated by METH in endothelial cells [23]. We tested whether TAAR-1 mediated endothelial dysfunction induced by METH in RRMECs and cultured OA by using EPPTB, a specific TAAR-1 antagonist. As shown in Figure 7a,b, EPPTB treatment effectively reversed the alterations of MMP-9 and syndecan-1 at protein (*p* < 0.001 for MMP-9 and *p* < 0.05 for syndecan-1, *n* = 3, Figure 7a) and mRNA levels (*p* < 0.05 for MMP-9 and *p* < 0.001 for syndecan-1, *n* = 5–6, Figure 7b) in METH-administered RRMECs, compared to cells administrated METH alone. In cultured OA, the increased protein level of MMP-9 and decreased protein level of syndecan-1 by METH administration were significantly reversed in OA incubated with METH in the presence of EPPTB (*p* < 0.05 for MMP-9 and syndecan-1, *n* = 3), compared to the METH-only administered group (Figure 7c).

**Figure 8:**
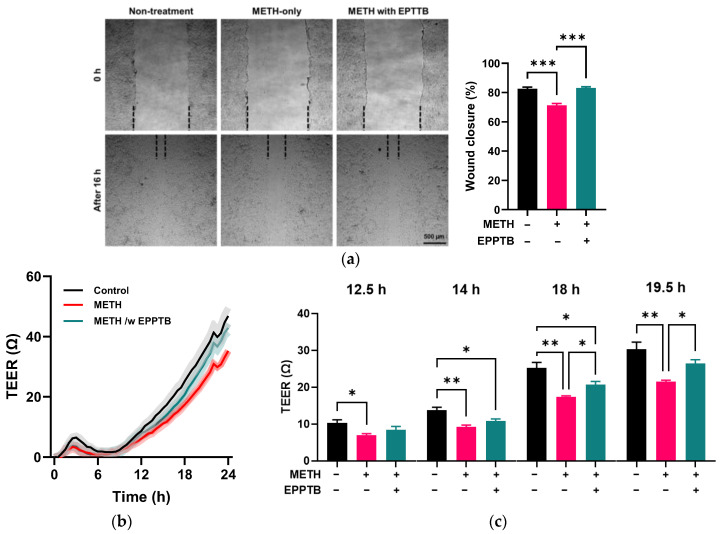
Effect of the TAAR-1 inhibitor, EPPTB, on METH-induced changes. (**a**) Representative images of scratched areas in RRMECs were captured using a microscope. The dashed line indicates the boundary of the cell monolayer. RRMEC migration (“wound closure”) was quantified by measuring the scratched area at 0 h and 16 h using ImageJ (*n* = 3, means ± SEM, *** *p* < 0.001). (**b**,**c**) Endothelial resistance in RRMECs incubated without or with METH in the absence or presence of EPPTB was continuously monitored using a TEER assay (*n* = 4, means ± SEM, * *p* < 0.05 and ** *p* < 0.01).

In addition, EPPTB treatment enhanced wound closure in METH-treated RRMECs (*p* < 0.001, *n* = 3, Figure 8a) compared to the METH-only group. The TEER assay (Figure 8b,c) showed that resistance began to change significantly at 12.5 h following METH administration, compared to the control group (*p* < 0.05, *n* = 6). However, in the presence of EPPTB, METH-treated cells did not show a significant difference compared to the control group until 14 h. By 18 h, EPPTB treatment significantly increased resistance in METH-administered RRMECs compared to those treated with METH alone. At 19.5 h after treatment, there were no significant changes in resistance between control vs. METH with EPPTB administration.

## 4. Discussion

METH promotes retinal angiogenesis and increases permeability by disrupting vascular homeostasis through oxidative stress, inflammation, and tight junction destabilization. METH upregulates vascular endothelial growth factor a (VEGFa), potentially through hypoxia-like responses and hypoxia inducible factor-1α (HIF-1α) activation [24], leading to excessive endothelial proliferation and neovascularization [23]. Simultaneously, METH compromises the BRB by downregulating tight junction proteins, inducing inflammatory cytokines like tumor necrosis factor-α and interleukin-6, and activating MMP-9, which degrades the extracellular matrix [23]. These combined effects contribute to vascular leakage, retinal edema, and pathological neovascularization, resembling changes seen in conditions like diabetic retinopathy, potentially exacerbating ischemic retinal injury.

In previous studies, we demonstrated that repeated METH administration induces retinal hypoxia and promotes aberrant angiogenesis, characterized by increased expression of HIF-1α and VEGFa signaling [24]. These molecular alterations were accompanied by the loss of critical endothelial surface molecules, including syndecan-1, which is essential for maintaining vascular homeostasis [2]. Although speculative, the observed increase in syndecan-1 mRNA may represent a compensatory response to protein loss mediated by MMP-9. The simultaneous activation of pro-angiogenic pathways and degradation of endothelial structural components suggests that METH exposure creates a maladaptive environment within the retinal vasculature—one that favors pathological vessel formation over stable, functional angiogenesis. This altered vascular phenotype may increase the susceptibility of the retina to ischemic injury, edema, and neovascular complications. Interestingly, while METH-induced HIF-1α/VEGFa activation enhances retinal endothelial cell proliferation [23], our current findings show a contrasting effect on another key component of angiogenesis—cell migration. Specifically, METH administration significantly suppressed the migratory capacity of retinal endothelial cells, which is a critical step for endothelial tip cell extension, sprouting, and directional elongation of new blood vessels [25]. This paradox highlights a potentially uncoupled angiogenic response, where endothelial cells proliferate but fail to organize and migrate properly, resulting in disorganized or incomplete vascular networks. Such dysregulated angiogenesis is a well-documented feature in retinal diseases like proliferative diabetic retinopathy and neovascular age-related macular degeneration [26].

The consequences of METH-induced endothelial pathology were functionally evident in our TEER assay, where administration of 2 µM METH, which is the average plasma concentration found in chronic METH users [1,27], exhibited delayed resistance development, reflecting impaired junctional integrity and reduced barrier function. Since endothelial barrier maturation is closely linked to cell–cell adhesion and cytoskeletal dynamics, the observed TEER delay further supports the idea that METH-induced endothelial pathology compromises not only migration but also the structural maturation of new blood vessels. Collectively, these results suggest that METH may impair physiological angiogenesis by altering the balance between endothelial proliferation, migration, and barrier formation. This dysregulation could contribute to the formation of leaky, immature vasculature, ultimately exacerbating retinal pathology under chronic METH exposure.

Syndecan-1 is abundantly expressed on the surface of endothelial cells and plays a central role in maintaining the integrity of the endothelial glycocalyx. Beyond its structural contributions, syndecan-1 is actively involved in coordinating integrin-mediated cell migration by regulating cell–matrix adhesion, intracellular signaling cascades, and actin cytoskeleton remodeling [20]. Loss or functional impairment of syndecan-1 disrupts these processes, leading to diminished integrin activation and impaired migratory responses in endothelial cells [20]. This mechanistic insight is particularly relevant to our findings: exposure to METH resulted in a significant reduction of syndecan-1 expression and migration in retinal endothelial cells.

MMP-9 is a proteolytic enzyme known to degrade various components of the extracellular matrix, including proteoglycans such as syndecan-1 [16]. Syndecan-1 contains cleavage sites for several MMPs, and MMPs have been shown to directly cleave recombinant syndecan-1 in vitro [28]. Previous studies have demonstrated that inhibition of MMPs in retinal endothelial cells can prevent the loss of syndecan-1 under hyperglycemic conditions [12], implicating MMPs in endothelial glycocalyx damage in diabetic retinopathy and other vascular diseases. In the present study, we found that treatment with recombinant MMP-9 significantly reduced syndecan-1 protein levels in retinal endothelial cells. Conversely, pharmacological inhibition of MMP activity restored syndecan-1 expression that was otherwise decreased by METH exposure, both in vitro and in cultured OA. These results strongly suggest that MMP-9 activity is important for METH-induced syndecan-1 shedding.

Moreover, the inhibition of MMPs not only preserved syndecan-1 levels but also restored endothelial function, as evidenced by improved cell migration and barrier integrity in TEER assays. These findings align with a prior report [20] highlighting the importance of syndecan-1 in integrin-mediated signaling and cytoskeletal organization during endothelial migration. Loss of syndecan-1 compromises cell–matrix interactions and integrin activation, leading to impaired endothelial dynamics essential for angiogenic sprouting and vascular remodeling. The observation that MMP inhibition reverses these functional impairments suggests a direct mechanistic link between syndecan-1 degradation and abnormal angiogenic responses. Given that MMP-9 is also elevated in other pathologic conditions such as inflammation, ischemia, and diabetes, our results suggest that MMP-9–mediated syndecan-1 loss could be a common pathway contributing to endothelial dysfunction across multiple retinal disease contexts. From a therapeutic standpoint, targeting MMP-9 offers a promising strategy to preserve endothelial glycocalyx integrity and restore normal angiogenic behavior. MMP inhibitors have already been explored in oncology and inflammatory diseases; however, their utility in retinal vascular diseases remains under-investigated. Localized or controlled delivery of MMP inhibitors could provide vascular protection without the systemic side effects observed in broader clinical trials.

TAAR-1 is a G-protein-coupled receptor that binds trace amines and also serves as a receptor for METH [21,22,23]. TAAR-1 is expressed in various tissues, including the vasculature, and has recently gained attention for its role in mediating the cellular effects of psychostimulants. In endothelial cells, activation of TAAR-1 by METH initiates a cascade of intracellular signaling events that culminate in the activation of HIF-1α and VEGFa, ultimately promoting endothelial cell proliferation [23]. These findings suggest that TAAR-1 is a key mediator of METH-induced endothelial responses and may contribute to vascular remodeling under pathological conditions. In the present study, we further investigated the downstream consequences of TAAR-1 activation in the context of METH-induced retinal vascular pathology. Specifically, we found that pharmacological inhibition of TAAR-1 attenuated the METH-induced upregulation of MMP-9 and preserved syndecan-1 protein levels in both retinal endothelial cells and OA. These results strongly suggest that TAAR-1 mediates METH-induced syndecan-1 degradation through the upregulation of MMP-9. This pathway provides a mechanistic link between METH exposure and loss of endothelial structural integrity, implicating TAAR-1 as a central regulator of METH-induced glycocalyx disruption. Furthermore, TAAR-1 inhibition restored endothelial cell migration and improved barrier function. These functional improvements reinforce the notion that TAAR-1 plays a pivotal role in mediating the broader spectrum of endothelial dysfunction observed with METH exposure, including impaired cell motility and compromised junctional integrity. Given that both migration and barrier formation are essential processes for angiogenesis and vascular homeostasis, our findings highlight TAAR-1 as a potential therapeutic target for preventing or reversing METH-induced retinal microvascular damage.

## 5. Conclusions

In conclusion, our study identifies a novel mechanistic pathway by which METH induces retinal endothelial dysfunction. We demonstrate that METH exposure leads to the degradation of syndecan-1—a critical component of the endothelial glycocalyx—via upregulation of MMP-9. This process is mediated through activation of TAAR-1, which initiates downstream signaling that not only enhances MMP-9 expression but also impairs endothelial migration and barrier integrity. Pharmacological inhibition of either MMPs or TAAR-1 effectively preserved syndecan-1 levels and restored endothelial function, underscoring the importance of this pathway in METH-induced retinal vascular pathology. These findings suggest that TAAR-1–MMP-9–syndecan-1 signaling may be a key driver of abnormal angiogenesis and vascular vulnerability in the retina. Targeting TAAR-1 or MMP-9 could represent a promising therapeutic strategy for preventing or ameliorating microvascular damage associated with METH exposure and potentially other ischemic or inflammatory retinal conditions.

## Figures and Tables

**Table 1:** Primer information.

Gene Symbol	Accession No.	Gene Name	Primer Pair (5′ to 3′)
Mapk1	NM_053842.2	Mitogen activated protein kinase 1	ATCTCAAGATCTGTGACTTTGG
			CTACATACTCTGTCAAGAACCC
Mmp2	NM_031054.2	Matrix metalloproteinase 2	CTGCAAGCAAGACATTGTC
			CGCCAAATAAACCGATCCT
Mmp9	NM_031055.2	Matrix metalloproteinase 9	CCAACCTTTACCAGCTACTC
			TTGTAGGGTCGGTTCTGA
Ppia	NM_017101.1	Peptidylprolyl isomerase A	TGTGGCCCTCCTACATAAA
			AGTAGGAGACTAACCACGTG
Sdc1	NM_013026.2	Syndecan 1	CCAAATCCGGACACCAAA
			GGGCACCAAACAGATAGTC

## Data Availability

Data will be made available upon request.

## Abbreviations

The following abbreviations are used in this manuscript:

ADAM	A disintegrin and metalloproteinases
BBB	Blood–brain barrier
BRB	Blood–retina barrier
EPPTB	N-(3-ethoxyphenyl)-4-(1-pyrrolidinyl)-3-(trifluoromethyl)benzamide)
HIF-1α	hypoxia inducible factor-1α
Mapk1	Mitogen activated protein kinase 1
METH	Methamphetamine
MMP	Matrix metalloproteinase
OA	Ophthalmic artery
PBS	Phosphate-buffered saline
Ppia	Peptidylprolyl isomerase A
RRMEC	Primary rat retinal microvascular endothelial cell
Sdc1	Syndecan 1
TEER	Transendothelial Electrical Resistance
TAAR-1	Trace amine-associated receptor-1
VEGFa	Vascular endothelial growth factor a
